# Unrecognized concomitant ventricular septal rupture and left ventricular aneurysm 10 months after myocardial infarction in a patient presenting with chronic heart failure

**DOI:** 10.1186/s12872-021-02360-4

**Published:** 2021-11-17

**Authors:** Masashi Kawamura, Osamu Monta, Kana Shibata, Yasushi Tsutsumi

**Affiliations:** Department of Cardiovascular Surgery, Fukui Cardiovascular Center, Shinbo 2-228, Fukui City, Fukui Prefecture 910-0833 Japan

**Keywords:** Ventricular septal rupture, Left ventricular aneurysm, Chronic heart failure

## Abstract

**Background:**

We report a rare case of concomitant inferior left ventricular aneurysm and ventricular septal rupture in a patient presenting with chronic heart failure.

**Case presentation:**

An 81-year-old man suffered from congestive heart failure. His symptoms were alleviated by medical management; however, heart failure symptoms continued according to the New York Heart Association Functional Classification III. Ten months after presentation, ventricular septal rupture was diagnosed using echocardiography. The left ventricular aneurysm was also complicated. Surgical repair of the ventricular septal rupture and left ventricular aneurysm was successfully performed. The ventricular septal rupture consisted of multiple holes, and the infarcted myocardium had already progressed to firm, fibrotic scar tissue. We closed the ventricular septal rupture with a small bovine pericardial patch and performed an aneurysmectomy with a liner technique.

**Conclusions:**

Cases of ventricular septal rupture can have various clinical scenarios, and treatment should be optimized for each patient, especially with respect to the timing of surgery.

## Background

Ventricular septal rupture (VSR) is a fatal mechanical complication of acute myocardial infarction (AMI). Hemodynamics usually deteriorate, and immediate treatment is needed. VSR patients who are treated medically have extremely high mortality at 30 days; however, the mortality rates of VSR remain high even when the patients are surgically managed [1]. Left ventricular aneurysm (LVA) is another fatal mechanical complication after AMI. It causes heart failure, ventricular tachycardia, and embolism. The benefits of surgical reconstruction for LVA have not been clearly proven [2], but their effectiveness was recently reported in selected patients [3–5]. Herein, we report a rare case of concomitant inferior left ventricular aneurysm and ventricular septal rupture in a patient presenting with chronic heart failure.

## Case presentation

**Fig. 1:**
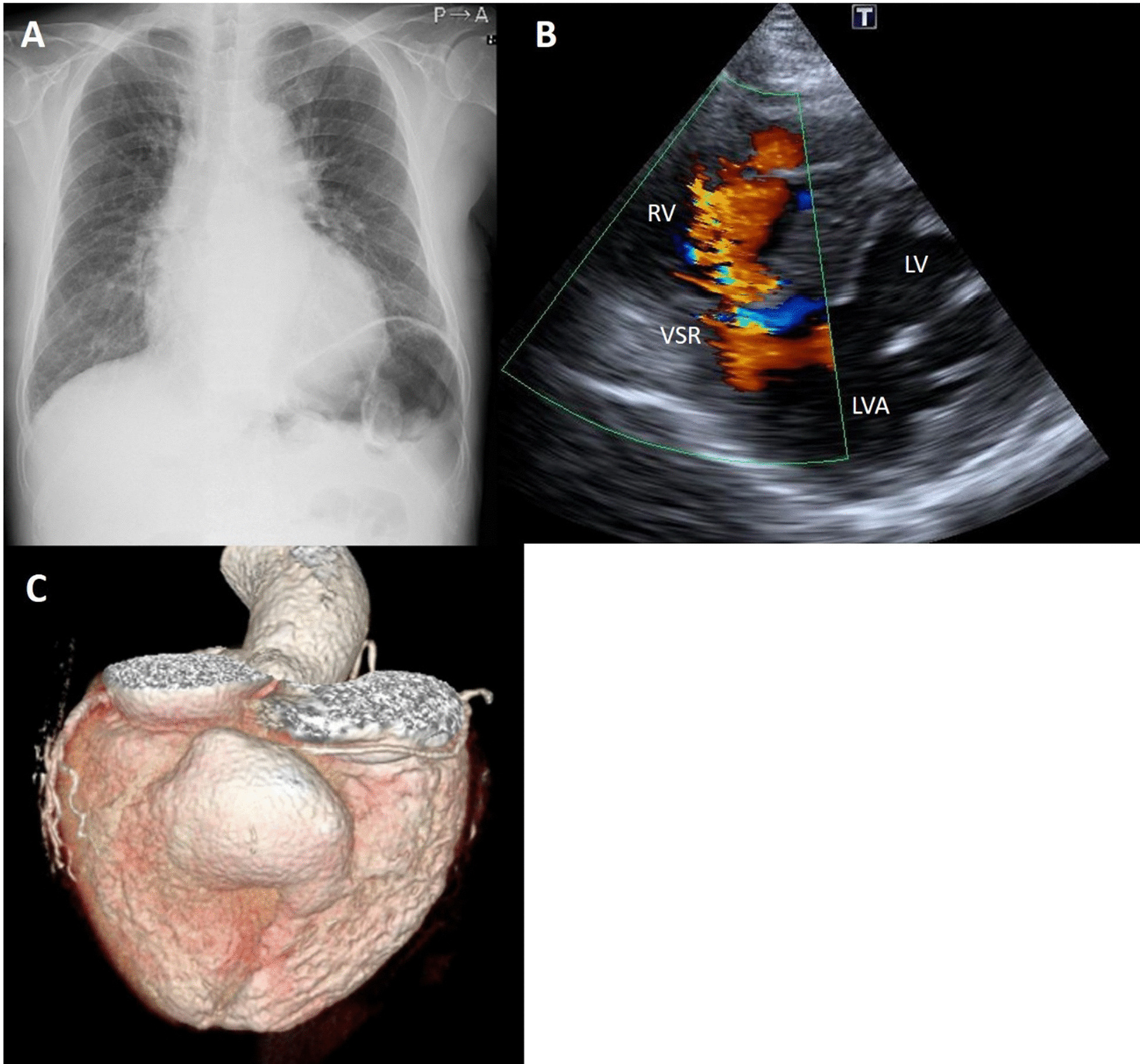
**a** Chest radiograph showed cardiomegaly and pulmonary edema. **b** Echocardiography at 10 month after presentation detected left-to-right shunt. **c** Three dimensional volume rendering image of cardiac computed tomography revealed an inferior LV aneurysm. *LV* left ventricle, *RV* right ventricle, *LVA* left ventricular aneurysm, *VSR* ventricular septal rupture

**Fig. 2:**
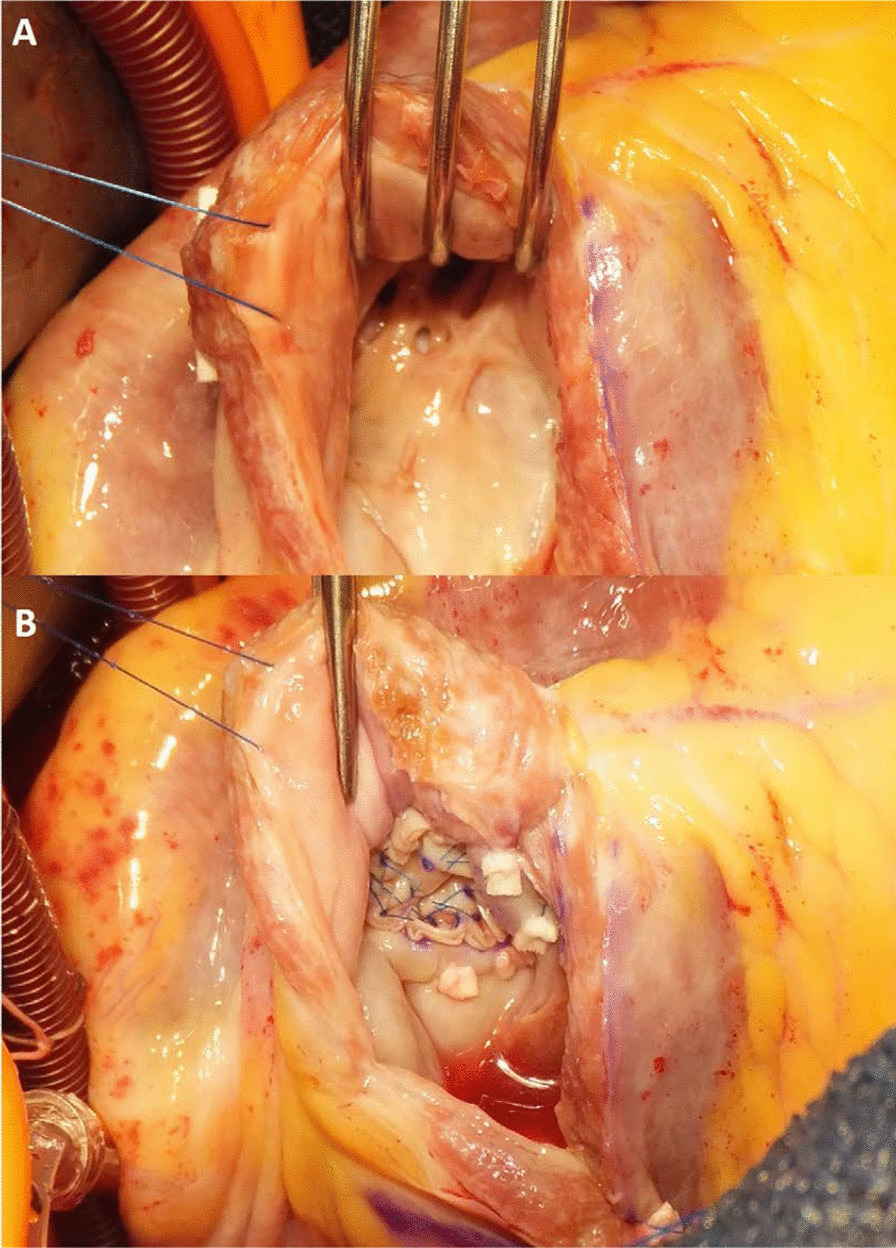
**a** The VSR site was found near the apex. The VSR consisted of multiple holes and the infarcted myocardium already turned the scar made of firm fibrosis tissue. **b** The VSR was closed with a 2.5 × 2.5 cm size of bovine pericardium patch

An 81-year-old diabetic man suffered from congestive heart failure. On admission, the electrocardiogram showed a Q wave in the III lead. Echocardiography demonstrated no apparent asynergy, and cardiac enzyme levels were not elevated. He also had epigastric pain, and coronary angiography was performed. There was significant stenosis of the right coronary artery (RCA) #2; the patient therefore underwent percutaneous coronary intervention on the RCA. His symptoms were alleviated with diuretics. However, heart failure symptoms continued according to the New York Heart Association Functional Classification III. Ten months after presentation, pulmonary edema on chest X-ray scan was exacerbated (Fig. 1a) and follow-up echocardiography revealed a shunt flow of ventricular septal rupture (Fig. 1b), moderate tricuspid regurgitation, and an estimated left ventricular ejection fraction (LVEF) of 35%. Cardiac computed tomography revealed an inferior left ventricular (LV) aneurysm (Fig. 1c). The dimensions of the LVA were 3.4 × 4.8 cm. Cardiac catheterization detected pulmonary high flow with an estimated Qp/Qs ratio of 3.5. Surgical treatment was indicated and the patient was referred to our hospital. The patient underwent repair of the ventricular septal rupture and liner repair of the inferior LVA. Cardiopulmonary bypass was established by ascending aorta cannulation and bicaval drainage. Intraoperatively, the VSR site was found near the apex when the inferior LVA was opened longitudinally. The VSR consisted of multiple holes (Fig. 2a), and the infarcted myocardium had already turned to scar tissue made of firm, fibrotic tissue. The VSR was closed with a 2.5 × 2.5 cm bovine pericardial patch that was fixed by 10 pairs of mattress sutures on the scar tissue around the VSR (Fig. 2b). The ventriculotomy was closed with two layers of 3–0 polypropylene mattress and running sutures with felt strips without any excision of the aneurysmal tissue. Tricuspid ring annuloplasty was also performed. A residual shunt was not found on intraoperative transesophageal echocardiography. Blood transfusions were performed intraoperatively. After surgery, the patient was extubated on the first postoperative day. He needed inotropic support for 4 days and stayed in the intensive care unit for 6 days. Medical management for heart failure included only diuretics. Postoperative transthoracic echocardiography on postoperative day 10 demonstrated an LVEF of 33% and no tricuspid regurgitation. The patient’s postoperative recovery was uneventful, and he was discharged from the rehabilitation facility on the 34th postoperative day (Table 1).

**Table 1:** Timeline

Timeline	
February 2020	First presentation of shortness of breath
March 2020	Coronary angiography revealed significant stenosis of the RCA and there was percutaneous coronary intervention for the stenosis
April–November 2020	New York Heart Association Functional Class III on medication with diuretics
Late-November–Early December 2020	Worsening dyspnea on exertion. Pulmonary edema on chest X-ray imaging, LVR on echocardiography, LVA on cardiac CT scan
Late December 2020	Successful repair of the VSR and LVA
January 2021	Patient discharged
Late April 2021	3 month- follow-up by the patient’s local cardiologist—no symptoms or complaints

## Discussion and conclusions

Recent studies have demonstrated that VSR is considered to complicate 0.17–0.31% of patients presenting with AMI [6–9], and the incidence of VSR has declined due to emergent reperfusion strategies for AMI [10]. LVA is another fatal mechanical complication of AMI and the incidence of LVA has also dropped due to the same reason. Therefore, VSR and LVA, especially inferior LVA as two mechanical complications of AMI in the same patient is rare.

Liner repair [11] and endoventricular patch plasty [12, 13] are the two major procedures for surgical treatment of LVA. Previous studies have reported that there is no difference in early or late outcomes [14–17]. The selection of the technique should be based on the anatomy of the patient’s aneurysm, such as its size, shape, scar dimension, and septal involvement [18]. In the present case, the LVA was relatively small. The aneurysm wall was not thin and the cavity volume was small. Therefore, we used the linear technique and obtained good results.

Our patient had a unique clinical scenario of VSR with symptoms of chronic heart failure but did not show cardiogenic shock. Some reports in the literature have demonstrated similar cases of VSR and LVA [10, 19, 20] or VSR [21] presenting with chronic heart failure. The uniqueness of the present case was that the patient survived for a longer period (approximately 10 months) with VSR and LVA without surgery, whereas other cases underwent surgical repair from 3 weeks to 2 months after the initial presentation. Although VSA and/or LVA are critical, these patients obtained good postoperative outcomes. Hemodynamics in VSR patients might depend on the size and location of the VSR, in addition to the size and location of the infarction, and VSR patients can show a wide range of clinical presentations.

The timing of VSR repair remains controversial. The American College of Cardiology guidelines suggest immediate surgery for VSR, regardless of the patient’s hemodynamic status [22]. In contrast, the European Society of Cardiology guidelines refer to delayed surgery in selected patients who respond to medical management [23]. Some reports demonstrated urgent or early surgery with a high mortality rate of 47–60% [24, 25], whereas delayed VSR repair after medical treatment was associated with a lower mortality rate of 15% [25]. Delayed surgery seems to be beneficial. In the present case, the occurrence of VSR was unknown. More than 10 months might have passed between the onset of myocardial infarction and surgery. The tissue around the VSR site was firm and stiff intraoperatively. We were able to stitch the scar tissue around the hole and close the hole using a small bovine pericardial patch. It was clearly technically easier than VSR repair in the acute phase. Every patient with VSR should undergo repair, while the timing of the repair should be considered based on the case of the patient, in terms of emergent surgery or elective surgery after medical optimization, etc. In addition, the percutaneous closure for VSR is becoming available and feasible, although there are some anatomical limitations, including the size, site, or shape of the VSR [26]. The percutaneous closure of the VSR in hemodynamically unstable patients as a bridge to surgery is ideal.

In conclusion, mechanical complications after AMI are rare. We encountered an extremely rare case of concomitant VSR and LV aneurysm presenting with chronic heart failure, and surgical repair of the VSR and LV aneurysm was successfully performed. VSR cases can have various clinical scenarios, and we should optimize treatment for each patient, especially with respect to timing of surgery.

## Data Availability

Not applicable.

## Abbreviations

VSR: Ventricular septal rupture
AMI: Acute myocardial infarction
LVA: Left ventricular aneurysm
RCA: Right coronary artery
LVEF: Left ventricular ejection fraction
